# Whose children matter? Multigenerational family complexity and late-life divorce in Sweden

**DOI:** 10.1007/s10433-025-00902-9

**Published:** 2026-01-23

**Authors:** Linda Kridahl, Ann-Zofie Duvander, Jani Turunen

**Affiliations:** 1 Department of Sociology, Stockholm University, Stockholm, Sweden; 2 Department of Humanities and Social Sciences, Mid Sweden University, Östersund, Sweden; 3 Social Sciences, Södertörn University, Stockholm, Sweden

**Keywords:** Late-life divorce, Family structure, Stepties, Older unions, Gender

## Abstract

In many Western countries, divorce among older age groups has slowly increased. One potential explanation is the increase in the diversity of family structures, such as a blend of joint children, stepchildren and stepgrandchildren. We investigate the association between multigenerational family complexity and late-life divorce in Sweden (60 +). Multigenerational family complexity includes a couple’s joint children/grandchildren and any children/grandchildren to whom one of the partners is a parent/grandparent, i.e. when the female partner, male partner or both partners have children/grandchildren from previous unions. Using Swedish register data, we find that couples with step relationships are more likely to divorce than those with only biological ties, that couples with only joint children or grandchildren have the lowest late-life divorce risk, and that couples with two sets of stepchildren face a higher risk than those with one. We also find that joint children in stepfamilies lower divorce risk among couples where the female partner has children from previous unions and for couples with both joint and step grandchildren, regardless of the stepchild’s parental lineage. Lineage patterns of family complexity in the third generation operate somewhat differently than those in the second generation do. We provide novel insights into how biological and stepties as well as maternal and paternal lineages across generations are related to divorce risk later in life.

**Supplementary Information:**

The online version contains supplementary material available at 10.1007/s10433-025-00902-9.

## Introduction

Divorce among older age groups has increased in Western countries in recent decades (Australian Institute of Family Studies 2023; Brown and Lin 2012, 2022; Solaz 2021; Tosi and van den Broek, 2020). One potential explanation, yet not examined, for the development of late-life divorce is the increasing diversity in family structures (Brown et al. 2021). Repartnering often brings together biological and nonbiological children, stepchildren and even stepgrandchildren. Stepfamilies often face additional challenges when children from different unions are brought into the new union by either the mother or the father, making the partner a stepparent (Stewart 2005). Compared with intact families, stepfamilies have been argued to be ‘incomplete institutions’, without the institutionalized solutions of everyday family life (Cherlin 1978; Coleman et al. 2015), including relationships with grandchildren (Gangong and Coleman 2012). Despite being common, stepfamilies still lack clear (gendered) norms and expectations.

Although research on stepfamilies and late-life divorce is limited, studies show that remarried older couples in the U.S. face higher divorce risk (Brown and Lin 2012), reflecting the adverse ‘repartner effect’ on couple stability found in the general population (Zahl-Olsen 2022). Related to family, Lin et al. (2018a, b) expected that couples aged 50 + with stepchildren would be more likely to divorce than couples with joint children and childless couples would be, but their US-based study did not confirm this. Since family dynamics and the meaning of step relationships may differ in other contexts, for example, in European Nordic countries, further research is needed.

These family dynamics in ageing families have also been linked to ties in the third generation; for example, grandchildren have been shown to decrease the risk of late-life divorce among older American and European couples (Brown et al., Alderotti et al. 2022), whereas stepgrandchildren have been shown to increase divorce risk, at least in the U.S. context (Brown et al. 2021). There are two main pathways to step-grandparenthood (Ganong and Coleman 2004; Steinbach and Silverstein 2020): when an adult child partners with someone who has children (i.e. inherited stepgrandparenthood) or when adult stepchildren have children (the focus of this study). While the first pathway often leads to larger and complex networks, both situations highlight how step relations may shape coupled stability. Moreover, having a joint child or grandchild can strengthen bonds and promote stability in ageing stepfamilies (Bildtgård et al. 2021; Schoeni et al. 2022; Stewart 2005). Taken together, these findings suggest that both the presence of joint or step relations and the length of the marriage need to be considered to fully understand the dynamics of late-life divorce in stepfamilies.

Using Swedish register data, this study examines the link between multigenerational family complexity and divorce from age 60 (cohorts 1930–1956), without making causal claims. The focus is on couples (first generation), their joint children (second generation) and grandchildren (third generation), as well as children/grandchildren from other partnerships, while accounting for both maternal and paternal lineages. Marital duration is also considered, as longer marriages are generally more stable and have more integrated family relationships. To capture these associations in a clear and transparent way, we rely on a deliberately narrow operationalization of family complexity. In this study, we study divorce following marriage and not cohabitation, and family complexity includes the presence of children and grandchildren from current or previous unions, whether followed by divorce, separation or widowhood, rather than marital history per se. The definition does not capture whether the (young or adult) children coreside with the parents or inherited step-grandparenthood and is, thus, a conservative definition of family complexity. Our definition limits the generalizability to all forms of complex family structures.

### Sweden as a context for studying late-life divorce and family complexity

Sweden’s family demographic history (e.g. Ohlsson-Wijk et al. 2020) makes it a valuable context for studying multigenerational family complexity and late-life divorce. It was among the earliest countries to embrace values linked with divorce, such as individualization, self-expression, and secularization, while weakening traditional family norms (Lesthaeghe 2014; Surkyn and Lesthaeghe 2004), alongside changes in gender roles and family norms (Goldscheider et al. 2015). Unlike in many other countries, separated fathers have remained actively involved with their children for decades, partly because of joint physical custody arrangements (Bergman and Hobson 2002). High female labour force participation and welfare support strengthen women’s economic independence and ability to support themselves after divorce, though their lifetime earnings and pensions remain lower than men’s (Stanfors 2017; Wetterberg 2013), improving their ability to support themselves after divorce, albeit with lower lifetime earnings and pensions than men (Statistics Sweden 2014a, 2014b, 2024).

While most earlier studies define late-life divorce from age 50 (Lin et al. 2018a, b; Alderotti et al. 2022), this study focuses on age 60 (Bildtgård and Öberg 2022; Kridahl and Kolk 2025; Kridahl et al. 2025), as this age better reflects demographic and social developments among the older population in Sweden. Age 60 often indicates a transition to retirement and increased vulnerability, whereas ages 50–59 are typically shaped by ongoing family responsibilities (including coresidential children) and labour force participation (Statistics Sweden 2020). In Sweden, divorce at age 60 + rose from 1.4 per 1000 marriages in 2000 to 2.6 in 2025 (Kridahl and Kolk 2025; Statistics Sweden 2025). The rise in late-life divorce occurred against the backdrop of historically high divorce rates during the 1960s–1990s, which stabilized or even declined after 2000 (Andersson and Kolk, 2015). Among women aged 60 born 1930–1956, divorce increased from 14 to 22% and never-married status from 6 to 19%, while marriage declined from 68 to 55%; men show similar trends (Statistics Sweden 2025). Over the same period, widowhood became less common. The share of women widowed at age 60 fell from 12 to 4%, with comparable declines among men (Statistics Sweden 2025). Concurrently, the median age at widowhood rose from about 70 to 79 for men and from 66 to 77 for women between 1970 and the late 2000s, while the median duration of widowhood fell from 17 to 12 years for women and stayed stable for men (Dabergott 2022). These shifts indicate that widowhood was more common in earlier cohorts but now mostly occurs after the childrearing years, making its role in stepfamily formation smaller. Sweden has also experienced high rates of repartnering and multiple partner fertility (Ohlsson-Wijk et al. 2020), resulting in a historically large share of individuals in stepfamilies. These family forms have also been prevalent among today’s older population, making Sweden a forerunner in this regard.

### Selection into different family compositions and late-life divorce

Studying divorce after age 60 inherently involves selection processes. First, there is a survivor selection (Fors et al. 2021): individuals who remain married later in life. These individuals are negatively selected for divorce proneness, as those who were more prone to divorce may have already experienced marital dissolution earlier in life. This survivor selection leaves a more stable pool of older married individuals. Second, divorced individuals, including multidivorces, often follow distinct trajectories that increase the risk of marital dissolution (Li and Penning 2023; Poortman and Lyngstad 2007; Zahl-Olsen 2022), including poorer health (Guner et al. 2018; Torvik et al. 2015), lower earnings (Kridahl et al. 2025; Schmauk and Kridahl 2024), more precarious positions in the labour market (Schmauk 2024), and personality traits linked to higher divorce risk (e.g. Spikic and Mortelmans 2021). Stepfamilies in later life may thus carry forward these selection factors, whether they are formed after divorce, separation, or widowhood. Couples with prior-union children who remain together until age 60 may be resilient but less integrated than couples with only joint children are. Some unions were formed recently, which may further weaken integration (compared with long-standing families with joint children). Older couples with both joint children and stepchildren may have formed in their reproductive years and been married longer than couples without joint children, while those with only stepchildren may reflect selection into low fertility, infertility and intentional childlessness (e.g. couples who chose not to have a joint child or unions between older men and much younger women).

The selection implies that the associations we observe are among a subset of more stable couples, while selection into family complexity highlights that prior life-course experiences are not random but are systematically related to divorce risk. To account for these dynamics, our analyses adjust for marital duration and differentiate between family configurations, providing a more nuanced basis for assessing the relationship between family complexity and late-life divorce.

## Research on stepfamilies and late-life divorce risk and study’s hypotheses

Research on ageing stepfamilies and late-life divorce is limited, particularly in the Swedish context, where most existing work is qualitative (Bildtgård et al. 2021; Bildtgård and Öberg 2022). Insights into challenges affecting late-life marital stability can therefore be drawn from studies of younger (step) families. Below, we review the relevant literature, highlight empirical directions, and conclude with six hypotheses. As we study divorce among older couples, the hypotheses take the perspective of the couples.

Living in a stepfamily may enrich life and provide support in difficult times, particularly later in life (Connidis 2020). However, research consistently shows that stepfamilies present unique challenges for all members, especially the couple (Ganong and Coleman 2004; Hobart 1991; van Eeden-Moorefield and Pasley 2013). Repartnered (older) couples not only face a higher divorce risk (“repartner effect”) (Kridahl and Kolk 2025; Raley and Sweeney 2020; Zahl-Olsen 2022), but as Beaujouan (2016) reported, this risk is higher among those who also formed stepfamilies than among couples with only joint children. The challenges are particularly pronounced when both partners have children from previous relationships (i.e. stepchildren). While this particular study does not consider age-specific divorce risk or distinguish older couples, it reveals that regardless of the partners’ age, the different responsibilities to “my” and “your” children may be conflictual, a situation that is not present in a family where both partners are the biological parents of all of the children.

Stepfamilies also encounter other challenges, such as bringing together two family cultures and individuals often at different stages in family life cycles. Both young and older parents alike tend to feel closer to their biological children than to stepchildren (Becker et al. 2013; Bildtgård and Öberg 2022; O’Connor and Boag 2010), and stepparenting is often experienced as more challenging than parenting one’s biological child (MacDonald and DeMaris 1995; van Eeden-Moorefield and Pasley 2013). Conflicts frequently arise around boundaries (Coleman et al. 2001), and loyalty tensions can place parents in a difficult position between their partner and children (Afifi 2003; Golish 2003), including among older couples (Lin et al. 2018a, b). In ageing stepfamilies, these challenges are compounded by care needs and potential disputes over financial transfers to children from previous unions (Raley and Sweeney 2020). Research has shown that intergenerational transfers of time and money occur less frequently in older stepfamilies than in biological families (Patterson et al. 2022) and that inheritance arrangements can introduce additional complexities (Jensen et al. 2019). Moreover, compared with biological parent–child ties, contact between older stepparents and adult stepchildren tends to decrease after a divorce (Noël-Miller 2013), highlighting the fragility of these relationships. Challenges in stepfamilies may particularly be pronounced when both partners bring children from previous unions. Although Fang et al. (2025) do not study divorce risk directly, they show that more complex stepfamily structures are perceived as less cohesive (see also Stewart 2005). Lower cohesion is likely to increase tension and, by extension, divorce risk, suggesting that couples with multiple sets of stepchildren face greater barriers to integration and greater family instability.

As the challenges of stepparenting extend into later life, these same complex family dynamics often carry over into grandparenthood. Although stepgrandparenthood is still a relatively underexplored topic (Chapman et al. 2016; Mongeon et al. 2024), the so-called step gap has also been found among stepgrandparent and stepgrandchild relationships. For example, Steinbach and Silverstein (2020) reported that stepgrandparents have less frequent contact and weaker emotional ties with stepgrandchildren than biological grandparents do, underscoring the difficulty of forging strong bonds in stepfamilies. Similarly, Sanner et al. (2018) reported that these ties typically dissolve when remarriage ends. These challenges in stepgrandfamily ties mirror broader patterns in marital stability; joint grandchildren reduce divorce risk among older American and European couples (Brown et al.; Alderotti et al. 2022), whereas stepgrandchildren increase the risk among American couples (Brown et al. 2021). On the basis of the previous findings, we formed our first two hypotheses:

### *Hypothesis 1*

Couples with stepchildren/stepgrandchildren have a higher risk of divorce than couples with only joint children/grandchildren do.

### *Hypothesis 2*

Couples with two sets of stepchildren/stepgrandchildren have a higher risk to divorce than couples with only one set of stepchildren/grandchildren are.

In stepfamilies with children from previous unions, a joint child can bring stepparents closer and help clarify family roles and strengthen bonds (the so-called concrete baby effect or bridge child) (Bildtgård et al. 2021; Fang et al. 2025; Ganong and Coleman 2017; Schoeni et al. 2022). Such children often stabilize repartnered unions (Sandström et al. 2014, however, though see Beaujouan 2016). Stepfamilies with joint children also report lower boundary ambiguity and higher cohesion than those without (Fang et al. 2025; Schoeni et al. 2022; Stewart 2005; Ward et al. 2009). Similarly, joint grandchildren have been found to strengthen the grandparent role and relationship stability (Brown et al. 2021), although no study has yet examined couples with both joint grandchildren and stepgrandchildren. We hypothesize the following:

### *Hypothesis 3*

Couples who have stepchildren/stepgrandchildren but no joint children/grandchildren have a higher risk of divorce than couples who have both joint and stepchildren/stepgrandchildren do.

Gendered dynamics also play a significant role in shaping stepfamily relationships. Women are traditionally seen as the main caregivers and kin keepers (Hornstra and Ivanova 2023; Kalmijn et al. 2019), which can lead to greater expectations for their involvement with their children. These expectations are likely to apply to her own children and, to a lesser extent, her partner’s (Cartwright 2012; Nielsen 1999). Stepmother–child relationships can be particularly strained because of the presence and influence of biological mothers (Ihinger-Tallman and Pasley 1997; Samzelius 2023). Children often see their mothers as central figures, and competition over resources (time, attention, financial support) can generate resentment towards a stepmother (Nielsen 1999), a phenomenon sometimes called the “stepmother trap” (Scholtz and Spies 2023).

Concurrently, unequal investment in stepfamilies often takes the form of “swapping families,” whereby parents reallocate time, money, and emotional support towards children in a new union at the expense of those from prior ones (Manning and Smock, 2000). Fathers, especially in the past, are particularly prone to this reallocation (a phenomenon called “recycling” family) (Arat and Poortman 2024; Furstenberg 1995, 1980; Furstenberg and Spanier 1984) and more strongly perceive their stepchildren as their own than stepmothers do (van Houdt 2023), resulting in lower contact frequency and weaker ties between them and their origin adult children (Kalmijn 2013, 2015), even after late-life divorce (Lin et al. 2022). Mothers with any stepchildren are less likely to live with any of their children than mothers of only biological children are (Seltzer et al. 2013), reflecting fathers’ distant relationships with their own children. Thus, the male partner takes on the role of a father in the new family, and his children are more of a threat to union stability and the established, e.g. routines and roles. His biological children may also destabilize older couples. For example, stepmothers (who often provide the majority of the caregiving and kin‐keeping) may be expected to invest in adult stepchildren despite weaker emotional bonds and greater logistical burdens (e.g. travel for visits and managing established living arrangements), which may create conflicts. Our fourth hypothesis is, therefore,

### *Hypothesis 4*

Divorce risk is higher among couples in which the man brings in children from a previous union into the union than among those in which a woman does.

Because of women’s role as family kin keepers, they also have closer ties with their grandchildren than men do (Hornstra and Ivanova 2023). Even following divorce, grandmothers continue to provide care at levels comparable to those of married grandmothers, whereas grandfathers do not (Amato et al. 1995). In stepfamilies, a woman’s intensive grandchild caregiving can draw time and emotional energy away from her partner, amplifying boundary ambiguity and creating loyalty conflicts that span generations. As a result, ties to her grandchildren may be stronger than those to his grandchildren (who are more at a distance), making women’s grandchildren more likely to negatively affect the couple’s relationship. Taken together, these gendered patterns of caregiving, resource allocation and intergenerational ties suggest that the origin of stepgrandchildren (woman or man) should differentially affect union stability. Our fifth hypothesis is as follows*:*

### *Hypothesis 5*

Divorce risk is higher among couples in which the woman brings in grandchildren from a previous union into the union than among those in which a man does.

Although stable couples are more likely to remain together into later life, as unstable unions often dissolve earlier, research from Europe and the U.S. shows that older couples generally face a reduced risk of divorce as marriages strengthen over time (Alderotti et al. 2022; Brown and Lin 2012; Kridahl and Kolk 2025; Kridahl et al. 2025; Lin et al. 2018a, b; MacDonald and DeMaris 1995). In stepfamilies, the timing of union formation (e.g. repartnering in midlife or later life), the duration of living as a stepfamily, and the children’s developmental stage at the time of repartnering (whether the children were minors or adults) often shape the integration process and can serve as a mitigating factor in the alleviation of stepties. Over time, stepfamily bonds tend to strengthen as routines form, boundary ambiguity declines, and shared rituals develop, which buffer conflict and reinforce commitment (Becker et al. 2013; Kalmijn 2013; Kalmijn et al. 2019). As a result, stepfamilies may increasingly resemble nuclear families (Hämäläinen et al. 2024), a process also seen in stepgrandparenthood (Chapman et al. 2018). Based on this reasoning, we propose the following hypothesis:

### *Hypothesis 6*

Divorce risk decreases among couples with stepchildren or stepgrandchildren as marital duration increases, similar to couples with only joint children.

## Data and Methods

### Study population and measure of late-life divorce

This study is based on extensive Swedish register data including all couples in the population who (1) were married (opposite-sex unions) when one or both of the partners were 59 years old (couples who divorced before age 59 are left censored) and (2) where at least one of the partners was born between 1930 and 1956. We thus observe late-life divorces from age 60 occurring in the period of 1990–2017 but follow the couples from the start of their marriage and observe any events that occur both before and after age 59 (e.g. child births). As the data include annual changes in civil status, we followed each couple until divorce (when at least one of the partners was 60 years or older), until they were right censored because of death, widowhood or out-migration, or until the end of the observation period in 2017, whichever occurred first (dependent variable, divorce, is time-varying). Moreover, the two criteria imply that there is a selection of couples in our population who remained married at least until age 59 and those who survived to older ages (by, e.g. socio-economic status, Fors et al. 2021). The study’s criteria also implied that not all cohorts were followed for an equally long period. Nonetheless, the cohorts were chosen so that it was possible to follow them over some years after age 60. Notably, we could only observe marriages, as it was not possible to trace cohabiting unions in the Swedish registers before 2011 if they did not have joint children (see sensitivity analyses at the end of the results section). Although cohabitation is common among younger and middle-aged individuals in Sweden, marriage remains the predominant form of union in later life, albeit with a declining prevalence. For example, 82% to 79% of women aged 60 (1951–56) and 86% to 81% of women aged 65 (1946–56) were married (Statistics Sweden 2025: own calculations).

### Measures of family complexity and adjusting variables

Using unique personal identification numbers from the register data, we linked each partner (first generation) to any Swedish‐born or Sweden‐residing child (second generation) or grandchild (third generation), from which we created two time-varying independent variables to capture family complexity. The first variable is *Family complexity in the 2nd generation*, which includes all combinations of (step) children, i.e. joint children and their children from their previous unions (regardless of divorce, separation and widowhood), and childless. The latter are included as a comparison because childlessness is an important later-life family configuration. The second variable, *Family complexity in the 3rd generation*, is similarly categorized, but the focus is on (step) grandchildren from the partners’ children. To facilitate interpretation, Table 1 presents all possible third-generation combinations derived from family complexity in the second generation. Table 2 displays the descriptive statistics for these variables when the unions enter the study period (i.e. when at least one of the partners becomes 59 years old). Notably, some of these groups are quite small; thus, caution should be taken when interpreting the results for these groups.

**Table 1:** Overview of all possible third-generation combinations derived from second-generation family complexity (childless excluded) ^1)^

	Family complexity in 3rd generation								
Family complexity in 2nd generation	Joint grandchildren	Joint grandchildren and she has grandchildren from previous unions	Joint grandchildren and he has grandchildren from previous unions	Joint grandchildren and both have grandchildren from previous unions	She has grandchildren from previous unions	He has grandchildren from previous unions	Both have grandchildren from previous unions	No grandchildren but couples has joint children	No grandchildren but couples has stepchildren
Joint children	X	N/A	N/A	N/A	N/A	N/A	N/A	X	N/A
Joint children and she has children from prev. unions	X	X	N/A	N/A	X	N/A	N/A	X	X
Joint children and he has children from prev. unions	X	N/A	X	N/A	N/A	X	N/A	X	X
Joint children and both have children from prev. unions	X	X	X	X	X	X	X	X	X
She has children from prev. unions	N/A	N/A	N/A	N/A	X	N/A	N/A	N/A	X
He has children from prev. unions	N/A	N/A	N/A	N/A	N/A	X	N/A	N/A	X
Both have children from prev. unions	N/A	N/A	N/A	N/A	X	X	X	N/A	X

^1)^ “X” = possible combination, “N/A” = not applicable

**Table 2:** Descriptive statistics of the studied couples at entry into the study period when at least one of the partners was 59 years old

		**%**
Family complexity 2nd	Joint children	54
generation	Joint children and she has children from previous unions	7
	Joint children and he has children from previous unions	8
	Joint children and both have children from previous unions	4
	She has children from previous unions	5
	He has children from previous unions	6
	Both have children from previous unions	11
	Childless	6
Family complexity 3rd	Joint grandchildren	30
generation	Joint grandchildren and she has grandchildren from previous unions	2
(Childless excluded)	Joint grandchildren and he has grandchildren from previous unions	7
	Joint grandchildren and both have grandchildren from previous unions	1
	She has grandchildren from previous unions	9
	He has grandchildren from previous unions	11
	Both have grandchildren from previous unions	7
	No grandchildren but couple has joint children	27
	No grandchildren but couple has step children	6
Period	1990–1999	22
	2000–2009	44
	2010–2017	34
Age composition	Partners are the same age or ± 2 years	40
	Woman is 3 or more years older	9
	Man is 3 or more years older	51
Educational	Both tertiary	17
composition	One tertiary and one secondary/primary	56
	Both secondary/primary	26
Marital duration	5 years or less	12
	6–10 years	5
	11–15 years	5
	16–20 years	7
	21–25 years	10
	26–30 years	14
	31 years or more	47
Country of birth	Both Swedish	81
	One born abroad	12
	Both born abroad	7
Working/retirement	Both retired	5
status	One working and one retired	17
	Both working	52
	One partly working and partly retired, one retired	3
	One partly working and partly retired, one working	11
	Both partly working and partly retired	1
	Both not working but have other source of income, one retired	3
	One working, both also have another source of income	8
	Both neither working nor retired but have another source of income	10
	Total population N	1,043,647

The models are adjusted for a set of common predictors of divorce. We make use of longitudinal characteristics or data to specify marital duration (time-varying from the year of marriage), calendar year (time-varying), age composition, educational composition (time-varying), country of birth and whether the partners are working or retired (time-varying) (Alderotti et al. 2022; Brown and Lin 2012; Chapman et al. 2016, 2018; Jalovaara 2003; Karraker and Latham 2015; Lin et al. 2018a, b; MacDonald and DeMaris 1995; Wilson and Waddoups 2002; Wu and Penning 1997, 2018). Descriptive statistics for these variables are displayed in Table 2. We do not adjust for the partner’s marital/cohabitation history because it is highly collinear with the family‐complexity measures and would substantially complicate the models, as this measure would require complete marriage and cohabitation history for each partner (i.e. her, his, and both partners’ history). Notably, this choice has implications for interpreting the observed associations. They may partly reflect prior union experiences, stepfamily dynamics, and selective processes that influence entry into different family trajectories. On the one hand, stepfamily dynamics and experiences from earlier unions may intensify the associations, making them appear stronger. On the other hand, selection into certain family trajectories may account for some of the associations, which would imply weaker effects than observed.

### Analytical strategy

This study employs complement log–log models with hazard ratios to estimate the risk of late-life divorce between 1990 and 2017. This analysis is suited for events recorded in discrete, continuous time intervals, such as divorces occurring within a given year (Allison 2010). Hazard ratios indicate an increase or decrease in divorce risk. For example, a ratio of 0.85 indicates a 15% lower risk of experiencing the event than the reference group does. The results include 99% confidence intervals; however, these are not tests of statistical significance, as the analysis covers the full population and not a sample. Two main models are estimated: the first includes second-generation family complexity and adjusts for year, age composition, marital duration, educational composition, partners’ country of birth and whether the couple’s working/retirement status. The second model focuses on third-generation family complexity and the same adjustment variables but includes only couples with children, excluding 6% who are childless. In additional models, we analyse the interactions between the two family complexity variables and marital duration. Notably, we analyse second- and third-generation complexity in separate models to keep them as simple as possible and to clearly distinguish how each dimension relates to late-life divorce. This strategy does not produce fully independent effects, as complexity in one generation may overlap with complexity in another, which should be kept in mind when interpreting the results.

### Regression results

The first hypothesis predicts that *couples with stepchildren/stepgrandchildren have a higher risk of divorce than those with only joint children/grandchildren (H1)*. To test this, we compare couples with only joint children/grandchildren to those with stepties, either alone or combined with joint ties. The results in Table 3 support the hypothesis for the second generation and show that couples with only joint children have the lowest risk of divorce. Most of the family complexity categories have a hazard ratio of 2 or closer to 3, indicating that these couples have a two- or threefold higher risk of divorce than couples with only joint children do. Table 4 reports the divorce risk by family complexity in the third generation. In accordance with the first hypothesis, the results show that couples who have only joint grandchildren have a lower risk of divorce than couples with all other family structures do.

**Table 3:** Risk of divorce at age 60 or older by family complexity in the second generation, adjusted complementing the log–log model, hazard ratios

		Risk of divorce	
		HR	99% C.I
Family complexity	Joint children	1	
2nd generation	Joint children and she has children from previous unions	1.52***	1.45–1.58
	Joint children and he has children from previous unions	2.10***	2.03–2.16
	Joint children and both have children from previous unions	2.73***	2.61–2.85
	She has children from previous unions	1.94***	1.85–2.02
	He has children from previous unions	2.04***	1.96–2.13
	Both have children from previous unions	2.86***	2.75–2.95
	Childless	0.99	0.94–1.03
Period	1990–1999	0.90***	0.86–0.96
	2000–2009	0.92***	0.88–0.95
	2010–2017 (ref)	1	
Duration	0–5	1.18***	1.13–1.23
	6–10	1.25***	1.20–1.30
	11–15	1.12***	1.08–1.17
	16–20	1.07***	1.03–1.11
	21–25	0.94***	0.91–0.98
	26–30	1	
	31 or longer		
Age composition	Partners are the same age or ± 2 years	0.66***	0.65–0.67
	Woman is 3 or more years older	0.87***	0.84–0.89
	Man is 3 or more years older (ref)	1	
Educational	Both tertiary	1.23***	1.20–1.26
composition	One tertiary and one secondary/primary	1.21***	1.19–1.24
	Both secondary/primary (ref)	1	
Country of birth	Both Swedish (ref)	1	
	One born abroad	1.88***	1.83–1.92
	Both born abroad	3.95***	3.85–4.06
Working/retirement	Both retired ^1)^ (ref)	1	
status	One working and one retired	2.27***	1.20–2.33
	Both working	2.09***	2.03–2.16
	One partly working and partly retired, one retired	1.43***	1.37–1.49
	One partly working and partly retired, one working	1.93***	1.86–2.00
	Both partly working and partly retired	1.16***	1.08–1.25
	Both not working but have another source of income, one retired	3.68***	3.54–3.84
	One working, both also have another source of income	3.57***	3.43–3.71
	Both neither working nor retired but have another source of income	4.66***	4.40–4.95
Total number of observations 8,516,603 (with 47,678 events of divorce)			
Note:^1)^ Individuals for whom 20% or more of their income is derived from their pension are defined as retired (in relation to their total income in a given year). Statistical significance levels: *** < 0.001

**Table 4:** Risk of divorce at age 60 or older by family complexity in the third generation, adjusted complementary log–log model among those with joint or stepchildren, hazard ratios

		Risk of divorce	
		HR	99% C.I
Family complexity	Joint grandchildren	1	
3rd generation	Joint grandchildren and she has grandchildren from previous unions	1.51***	1.36–1.66
	Joint grandchildren and he has grandchildren from previous unions	1.16***	1.10–1.23
	Joint grandchildren and both have grandchildren from previous unions	1.99***	1.74–2.28
	She has grandchildren from previous unions	2.04***	1.89–2.20
	He has grandchildren from previous unions	2.78***	2.63–2.95
	Both have grandchildren from previous unions	2.54***	2.23–2.89
	No grandchildren but couple has joint children	1.61***	1.54–1.68
	No grandchildren but couple has stepchildren	3.82***	3.57–4.09
Total number of observations 7,992,904 (with 451,888 events of divorce)			
Notes: Model adjusted for year, age composition, marital duration, educational composition and working/retirement status. Statistical significance levels: *** < 0.001			

The second hypothesis, that *couples with two sets of stepchildren/stepgrandchildren have a higher risk of divorce than couples with only one set of stepchildren/grandchildren,* is examined by rerunning the models in Table 3 and Table 4, with both having children/grandchildren as the reference. Overall, couples in which both partners had stepchildren or stepgrandchildren were consistently associated with the highest divorce risk, with all other family configurations facing a 5–45% lower risk, thereby supporting the hypothesis (joint + his children HR 0.76, 99%-C.I. 0.76–0.80; joint + her children HR 0.55, 99%-C.I. 0.52–0.59; his children HR 0.72, 99%-C.I. 0.69–0.75; her children HR 0.68, 99%-C.I. 0.65–0.71; joint + his grandchildren HR 0.61, 99%-C.I. 0.55–0.67; joint + her grandchildren HR 0.77, 99%-C.I. 0.69–0.87; his grandchildren HR 0.95, 99%-C.I. 0.89–1.01; and her grandchildren HR 0.72, 99%-C.I. 0.66–0.77).

The third hypothesis, that *couples who have stepchildren/stepgrandchildren but no joint children/grandchildren have a higher risk of divorce than those who have both joint and stepchildren/stepgrandchildren (H3),* is tested by also rerunning the model in Table 3 with a new reference category by gender. The results show that couples where only the female partner has children from a previous union have a 22% higher risk of divorce than couples with joint children, where the female partner has children (HR 0.78, 99%-C.I. 0.74–0.83). We observe a similar tendency among couples who have joint children and where both have children from previous unions (HR 0.95, 99%-C.I. 0.93–1.01). In contrast, among couples where the male partner has children from a previous union, the presence of joint children does not seem to matter for divorce risk (HR 0.97, 99%-C.I. 0.93–1.02 for the latter group of men). To test this hypothesis in the third generation, we conducted three comparisons by rerunning the model in Table 4. We first compare couples where only the female partner has grandchildren from a previous union (HR 1.45, 99%-C.I. 1.34–1.56) to couples with joint grandchildren, where the female partner has grandchildren from a previous union (reference). We thereafter perform the same type of comparisons (1) between couples where only the male partner has grandchildren from a previous union (HR 2.25, 99%-C.I. 2.34–2.57) and couples with joint grandchildren, where the male partner has grandchildren from a previous union (reference), and (2) between couples where both partners have grandchildren from previous unions (HR 1.46, 99%-C.I. 1.32–1.61) and couples with joint grandchildren, where both partners have grandchildren from a previous union (reference). These findings support our hypothesis across all three comparisons; couples without joint grandchildren have a higher risk of divorce than those with joint grandchildren.

The fourth hypothesis is that *divorce risk is greater among couples in which the man brings in children into the union than among those in which a woman does (H4)*. To address this hypothesis, we analyse a model (Table 3) in which couples who have joint children and who have children from previous unions are compared to couples who also have joint children but who have children from previous unions. We perform the same procedure for couples who have only stepchildren. The results show that among couples who have joint children, when the male partner also has children from a previous union, the couple has a 37% higher risk of divorce than when the female partner has children from a previous union (99%-C.I. 1.29–1.47). Among couples without joint children, the results indicate that there is a 5% greater risk of divorce when the male partner has children than when the female partner has children from a previous union (99%-C.I. 0.88–1.01). However, the slightly overlapping confidence intervals suggest that the results should be interpreted with caution and that the observed sex difference in divorce risk between these two groups may not be robust. Nevertheless, both tests support the hypothesis that the male partner’s children increase the risk of divorce in contrast to the female partner’s children.

The fifth hypothesis posits that grandchildren from the female partner impact family life and divorce risk more significantly than those from the male partner do, as his grandchildren are more absent. We hypothesize that *divorce risk is higher among couples in which the woman brings in grandchildren into the union than among those in which a man does (H5)*. This hypothesis is tested in two ways (model as in Table 4, but we change the reference category). First, couples with joint grandchildren for which the female partner has grandchildren from a previous union are compared to couples for which the male partner has grandchildren from a previous union. The results are in accordance with the hypothesis and indicate that couples where the female partner has grandchildren from a previous union have a 26% higher risk of divorce (99%-C.I. 1.17–1.37). Second, we compare couples where the female partner has grandchildren from a previous union with couples where the male partner has grandchildren from a previous union. In contrast with the hypothesis, the results show that couples in which the male partner has grandchildren have a 33% higher risk of divorce (99%–C.I. 1.28–1.38).

**Fig. 1:**
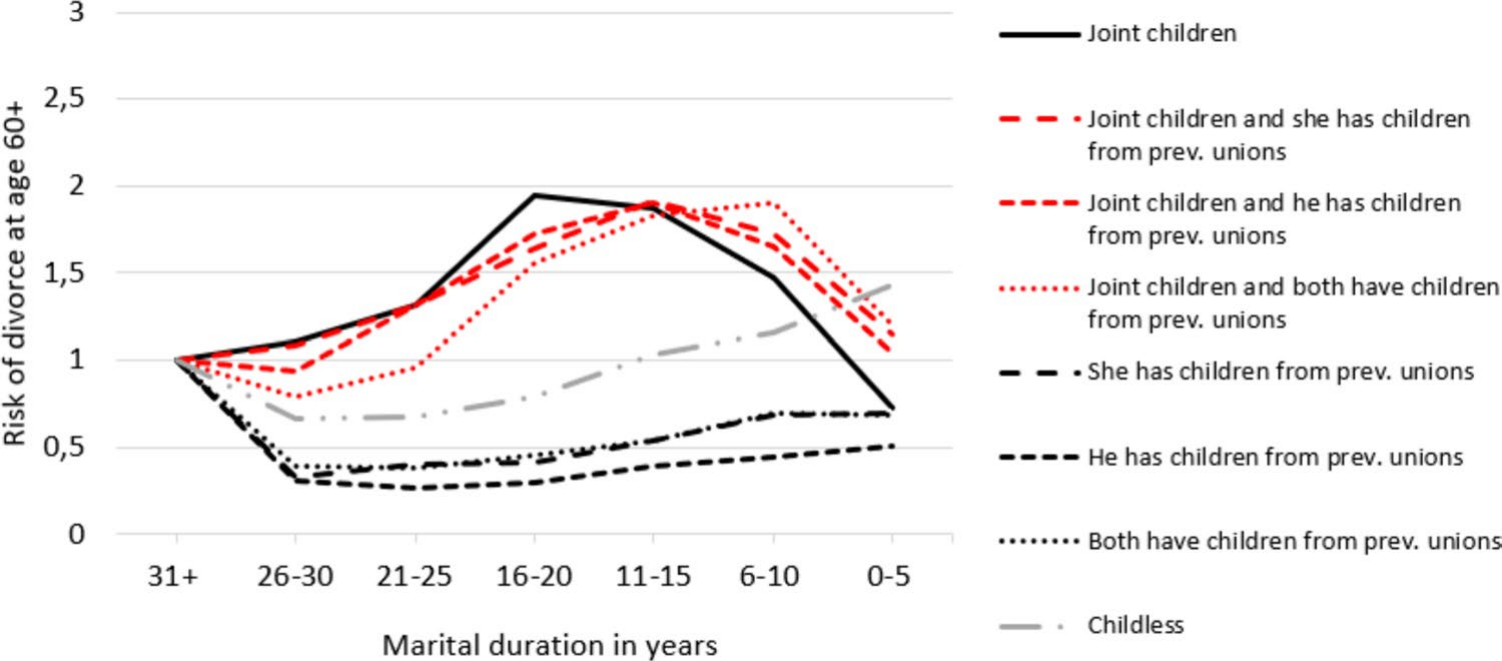
Risk of divorce at age 60 or older by family complexity in the 2nd generation interacted with marital duration, adjusted complementary log–log model, hazard ratios. *Notes*: Model adjusted for year, age composition, marital duration, educational composition and working/retirement status. Reference: Marital duration 31 + within each family composition. Corresponding Table 1 in Appendix

**Fig. 2:**
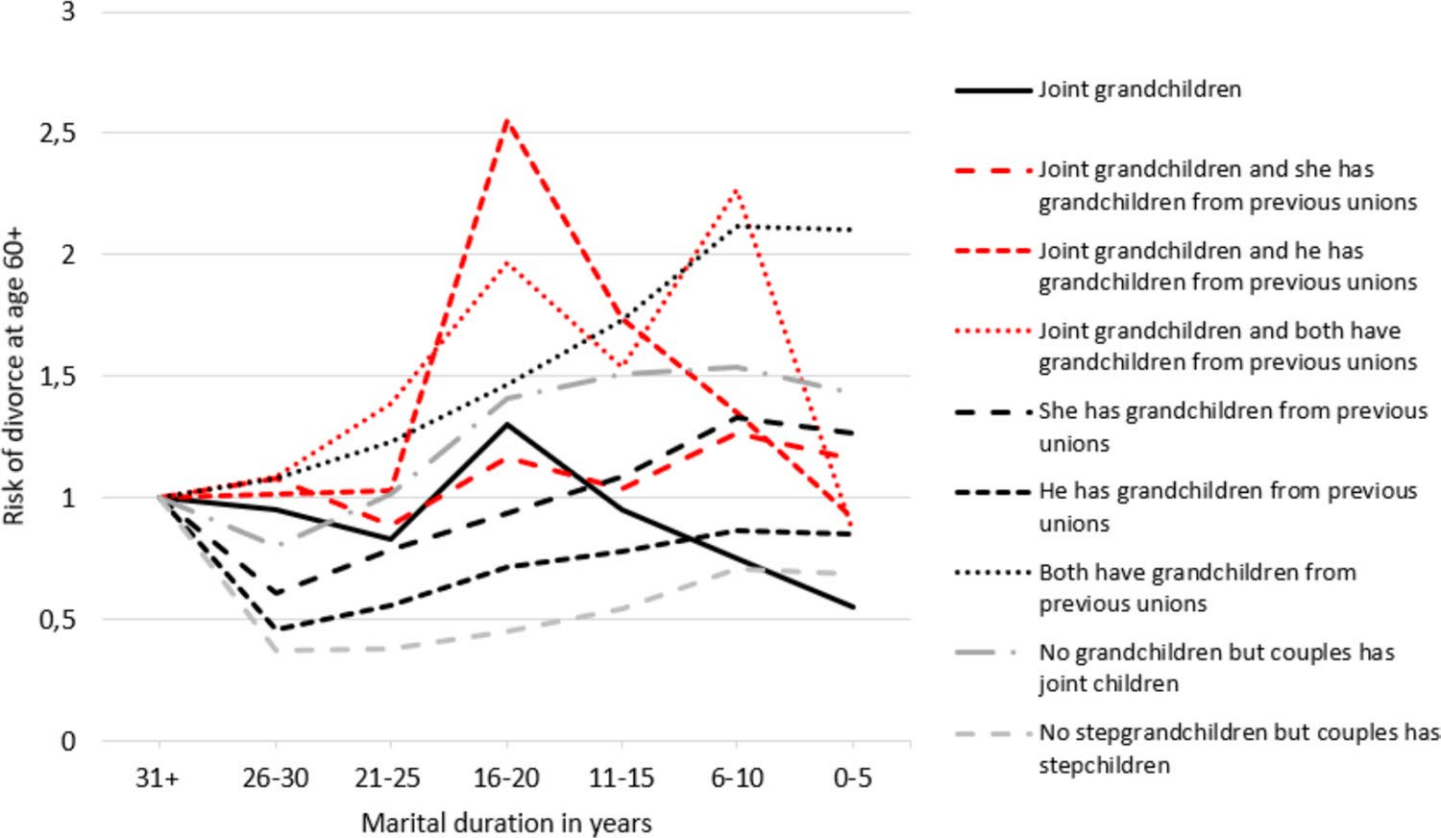
Risk of divorce at age 60 or older by family complexity in the 3rd generation interacted with marital duration, adjusted complementary log–log model, hazard ratios. *Notes*: Model adjusted for year, age composition, marital duration, educational composition and working/retirement status. Reference: Marital duration 31 + within each family composition. Corresponding Table 2 in Appendix

Next, we address the sixth hypothesis regarding the role of marital duration on divorce risk, i.e. that *divorce risk decreases among couples with stepchildren or stepgrandchildren as marital duration increases, similar to couples with only joint children (H6)*, by interacting the family complexity categories (in both the second and third generation) with marital duration in fully adjusted models (as in Tables 3 and 4). Figures 1 and 2 (with corresponding Tables 1 and 2 in the Appendix) show re-estimated hazard ratios for each category of family complexity with “marital duration 31 + years” as the reference group. Figure 1 shows that the pattern for all couples with joint children and stepchildren is an inverted U-shaped curve, following the curve for couples with only joint children. However, the peak of divorce risk is different for these groups, possibly because of the varying durations of union before marrying. In contrast, couples with only stepchildren have a slightly U-shaped curve, indicating that marital duration does not mitigate divorce risk in the same way. We conclude that couples with joint children follow similar paths of declining divorce risk over time, whereas this pattern does not hold for couples with only stepchildren.

Figure 2 (Table 2 in Appendix) shows that couples with joint and stepgrandchildren display an inverted U-shaped risk curve, peaking at intermediate durations before declining, whereas couples with only stepgrandchildren remain at an elevated risk across most durations, with only modest declines at 30 + years. Thus, H6 is only partially supported: while divorce risks diminish with longer marital duration, not all stepfamily couples converge towards the stability of those with only joint grandchildren. Interpreting marital duration remains complex, as many couples cohabit for substantial periods before marriage, making marital length an incomplete measure of relationship duration. Concurrently, censoring and selection effects may shape the patterns, since those who remain married longer are likely a more selective group.

### Sensitivity analyses

To check the robustness of our findings, we conducted additional analyses. First, we re-estimated the models in Tables 3–4 using only couples with complex family structures (childless couples are excluded). The results were consistent with those of the main models; however, most of the associations became stronger. Second, we included couples who married after age 59, but the results remained similar to those presented here, as few couples married after age 59. Third, we conducted an analysis including cohabiting couples at age 59 by using the dwelling register (available from 2011), limiting the analysis to 2012–2017. Interaction models indicated that compared with married couples, cohabiting couples had an overall greater risk of separating in all family complexity categories, including family complexity in the third generation. However, the hazard ratios were much higher, reflecting the rather small proportion of older couples who cohabited and separated during the short period of 2012–2017. To further analyse whether the association was sustained for couples whose partners were older than 65, we restricted the population to couples who were married when at least one of them was aged 64 and who were at risk of divorce from the age of 65; the associations were stronger for all categories of family complexity. Finally, we restricted the models to couples who were married for more than 20 years, which showed similar but stronger associations.

## Discussion

This study examined how multigenerational family complexity influenced the risk of divorce from age 60 in Sweden, including maternal and paternal lineage and marital duration. It took a novel couple-based approach with longitudinal register data. By doing so, it contributes to filling a gap in the literature on late-life couple stability and offers new insights into how step‐ and parent/grandparent ties may shape marital outcomes. While we did not capture all nuances of complexity or analyse second- and third-generation complexity simultaneously, this study offers an important step towards clarifying the role of family complexity in late-life marital trajectories.

In line with the first hypothesis, we found that couples with only joint children had the lowest risk of late-life divorce, supporting Cherlin’s (1978) argument of stepfamilies as incomplete institutions without clear guidelines. Thus, family complexity may create challenges in later life when couples rely on kin networks for emotional and instrumental support. Similarly, we also reported that having only joint grandchildren reduced the risk of late-life divorce, whereas having stepgrandchildren increased the risk, mirroring previous research on older European and American couples (Brown et al. 2021; Alderotti et al. 2022). These findings also align with the ‘repartner effect’, whereby repartnered couples face a higher risk of dissolution (Kridahl and Kolk 2025; Zahl-Olsen 2022). This interpretation, however, is tentative since our analysis did not account for partners’ marital history, and it is possible that, e.g. couples with only joint children or childless couples have been married before.

In line with our second hypothesis, couples in which both partners had stepchildren or step-grandchildren consistently displayed the highest divorce risk. This finding echoes evidence that complex stepfamilies are generally less cohesive (Fang et al. 2025; Stewart 2005). Larger family complexities, where multiple sets of step-ties intersect, likely amplify boundary ambiguities, loyalty conflicts, and competing obligations, making integration particularly difficult, also among older couples. This underlines that in later life, when couples rely more heavily on family support and stability, the challenges of managing multiple sets of step-ties may place older adults at particular risk of relationship strain and dissolution.

Additionally, the study found that couples with both joint and stepchildren or grandchildren had a lower divorce risk than those in stepfamilies without joint offspring did, except when the male partner brought children from a previous union. These findings support the idea that joint offspring function as ‘bridge’ or ‘concrete baby’ between stepties, fostering cohesion and buffering against dissolution (Bildtgård et al. 2021; Ganong and Coleman 2017; Schoeni et al. 2022). This stabilizing effect is also consistent with earlier research showing that stepfamilies with joint children have clearer boundaries and stronger cohesion than those without (Fang et al. 2025; Stewart 2005; Ward et al. 2009). For couples aged 60 + , sharing a grandchild may be especially meaningful as a joint project that may strengthen the relationship at a life stage when mutual goals and companionship become especially important (Brown et al. 2021).

In accordance with the fourth gender-specific hypothesis, we found that couples with stepmothers had a higher divorce risk (Nielsen 1999; Scholtz and Spies 2023). More precisely, divorce risk was lower when only the female partner had children from previous unions, compared with when the male partner did or when both partners did. This aligns with research showing that women are often viewed as primary caregivers and kin‐keepers (Hornstra and Ivanova 2023; Kalmijn et al. 2019), creating greater pressure on stepmothers to invest in both their own and their partner’s children (Cartwright 2012; Nielsen 1999). Such expectations can strain stepmother‐child relationships, as competition over time, attention, and resources can generate resentment (Scholtz and Spies 2023). Fathers tend to reallocate emotional and financial support towards children in a new union that can weaken ties with their adult children from prior unions (Manning and Smock, 2000; Arat and Poortman 2024; Kalmijn 2013). Despite these potential explanations, the study does not comprehensively explore how gendered stepfamily dynamics, prior union experiences, and selection processes shape the risk of late-life divorce.

Consistent with the fifth hypothesis, couples with joint grandchildren faced the highest divorce risk when the woman also had grandchildren from previous unions. Potentially, men’s weaker attachment to children, especially grandchildren, after separation means that the male partner’s ties to grandchildren are weaker than the female partner’s ties (gender differences in ties see e.g. Ahrons 2007; Hornstra et al. 2020; Hornstra and Ivanova 2023). Therefore, his grandchildren may not be perceived as a threat to the union. Concurrently, grandmothers are generally more engaged, which may create tensions in the union in cases where the stepgrandfather is not equally engaged in either their joint or her grandchildren. In contrast, when the stepgrandchildren belong to the male partner, the woman may engage more, since she is already more involved in the joint grandchildren (than he is), thereby reducing tensions between the partners. For couples with only stepchildren, divorce risk was lower when the female partner had grandchildren from previous unions, mirroring the second-generation pattern. This is likely explained by the stronger family ties mothers and grandmothers maintain, which often shape kin relationships more than those of fathers and grandfathers.

Moreover, as stepfamilies progress, their interpersonal bonds generally strengthen, leading to similarities with biological nuclear families in the context of stepgrandparenthood (Chapman et al. 2016, 2018; Hämäläinen et al. 2024). In support of our last hypothesis, we found that couples with joint and stepchildren/stepgrandchildren had a declining divorce risk over marital duration after some years of marriage. However, this was not the case for couples with only stepchildren/stepgrandchildren, either in the second or third generation. These couples are unique and quite selective in many aspects, and there are likely substantial differences between couples who have been married for a short duration and those who have maintained their marriage for several decades. Future research may need to disentangle these selection processes within and across these family structures, as this was out of the study’s scope.

As the study focuses on individuals aged 60 and older, the findings reflect the specific context of later life, i.e. retirement, health, and grandparenthood. Younger couples often face different circumstances, and future research could explore the role of family complexity on divorce across different age groups. Our results are shaped not only by age, but also by time. With increasing gender equality, gendered patterns may fade, and stepfamilies may better reflect cultural norms and become less challenging. Thus, the study may capture a transitional phase in both family life and broader societal change.

## Limitations and future research

This study has several limitations. First, couples may have separated before the official divorce date, potentially biasing timing. However, under the Swedish Marriage Act (1987:230), couples without children under 16 who mutually agree can divorce quickly, which is not uncommon within weeks, partly due to the no-fault grounds for divorce (Sandström, 2011). Second, the registers do not capture whether the couple had a relationship with all (step)children and (step)grandchildren or whether they coresided. Although young children are registered at one parent’s residence, joint custody is common in Sweden, with children often alternating weekly between parents, making it difficult to determine whether a child actually coresides with the parents. It is also very uncommon for adult children to live with their parents at this life stage in Sweden (Larsson 2007). Third, we include only the partner’s stepchildren and not their children’s own repartnering or multipartner childbearing (inherited stepgrandparenthood) (e.g. Ganong and Coleman 2017). This approach quickly increases family complexity and complicates interpretation. Fourth, our analysis does not account for the age of the child or life stage at union formation. However, this poses challenges, as there is no clear choice of which child’s age to use in stepfamilies. Additionally, children’s birth dates/age often do not coincide with union formation, making age endogenous to both repartnering timing and divorce risk. Finally, stepfamily categories correlate with age and life-course events, introducing collinearity, especially when marital duration is included. Fifth, we analysed second- and third-generation complexity in separate models, which means that the coefficients do not fully reflect independent effects. This may partly explain why some results, especially for grandchildren, diverge from our sixth hypothesis. Thus, these findings should be interpreted with this methodological choice in mind. Sixth, the analysis omitted key predictors of divorce, such as relationship quality, although it included many couple-level predictors that are important indicators of or proxies for different aspects of the relationship, such as couple stability and homogeneity.

Together with previous research, the study’s findings highlight how much remains to be understood about the drivers of late-life divorce and underline the need for continued investigation into this growing phenomenon (see also Carr and Utz, 2020). We did not explore pathways to stepgrandparenthood and complex families (Chapman et al. 2016), which would illuminate the significance of the timing of life events in the development of steprelationships on late-life divorce. Future studies would benefit from qualitative approaches, including postdivorce interviews with partners, children, stepchildren, and grandchildren, to deepen understanding of stepfamily dynamics (see, e.g. Koren et al. 2022; Samzelius 2023). Moreover, a potential consequence of late-life divorce of ageing stepfamilies is that stepparents often lose bonds with stepchildren and stepgrandchildren (Ganong and Coleman 2017), which may impact their social networks and quality of life; this highlights the need for further research on multigenerational stepties (e.g. Sanner et al. 2018). Finally, while this study focussed on Sweden, this does not mean that the role of complex family structures in later-life divorce is unique to Sweden. Similar demographic trends in Western Europe and the US warrant cross-national investigations to assess the broader relevance of these findings.

## Supplementary Information

Below is the link to the electronic supplementary material.

Supplementary file1 (DOCX 22 KB)

## Data Availability

The data supporting our study's results are derived from Swedish national administrative registers maintained by Statistics Sweden. More precisely, individual-level register data accessed through Stockholm University (Forte 2016–07115, "AgeingWell"). Access to these data is restricted due to confidentiality requirements. However, qualified researchers may order data and request access by contacting Statistics Sweden through their official website: https://www.scb.se/en/services/.
