# Supplementary material for: Whose children matter? Multigenerational family complexity and late-life divorce in Sweden
Source: Eur J Ageing. 2026 Jan 23;23(1):6. doi: 10.1007/s10433-025-00902-9 (PMC12847515; doi:10.1007/s10433-025-00902-9)
Supplement: Supplementary file 1 — Supplementary file1 (DOCX 22 KB) [file 10433_2025_902_MOESM1_ESM.docx]

**Appendix**

Table 1. The risk of divorce at age 60 or older by family complexity in the 2^nd^ generation interacted with marital duration, complementary log-log adjusted model, hazard ratios (reference marital duration within each family complexity category: 31+ years).

|  | Family complexity 2^nd^ generation | | | | | | | | | | | | | | | | | | | | | | | | |  |
| --- | --- | --- | --- | --- | --- | --- | --- | --- | --- | --- | --- | --- | --- | --- | --- | --- | --- | --- | --- | --- | --- | --- | --- | --- | --- | --- |
|  | Joint children | | Joint children and she has children from previous unions | | Joint children and he has children from previous unions | | Joint children and both have children from previous unions | | | She has children from previous unions | | | | He has children from previous unions | | | | Both have children from previous unions | | | | Childless | | | |  |
| Marital duration | HR | 99% C.I. | HR | 99% C.I. | HR | 99% C.I. | HR | 99% C.I. | | HR | | 99% C.I. | | HR | | 99% C.I. | | HR | | 99% C.I. | | HR | | 99% C.I. | |  |
| 31+ years | 1 |  | 1 |  | 1 |  | 1 |  | | 1 | |  | | 1 | |  | | 1 | |  | | 1 | |  | |  |
| 26-30 years | 1.10** | 1.04-1.16 | 1.08 | 0.94-1.24 | 0.94 | 0.84-1.04 | 0.79 | 0.65-0.97 | | 0.33*** | | 0.27-0.41 | | 0.31*** | | 0.25-0.37 | | 0.39*** | | 0.33-0.45 | | 0.66*** | | 0.57-0.78 | |  |
| 21-25 years | 1.32*** | 1.24-1.41 | 1.32*** | 1.14-1.52 | 1.32*** | 1.19-1.46 | 0.96*** | 0.80-1.16 | | 0.40*** | | 0.33-0.48 | | 0.27*** | | 0.22-0.32 | | 0.38*** | | 0.33-0.43 | | 0.67 | | 0.55-0.82 | |  |
| 16-20 years | 1.95*** | 1.76-2.15 | 1.64*** | 1.38-1.96 | 1.72*** | 1.55-1.92 | 1.56*** | 1.31-1.86 | | 0.41*** | | 0.35-0.49 | | 0.30*** | | 0.25-0.35 | | 0.45*** | | 0.40-0.50 | | 0.79 | | 0.63-1.00 | |  |
| 11-15 years | 1.87*** | 1.60-2.19 | 1.91*** | 1.53-2.38 | 1.90*** | 1.69-2.14 | 1.83*** | 1.52-2.21 | | 0.54*** | | 0.46-0.63 | | 0.39*** | | 0.34-0.45 | | 0.54*** | | 0.48-0.59 | | 1.03** | | 0.82-1.29 | |  |
| 6-10 years | 1.47*** | 1.18-1.84 | 1.72*** | 1.28-2.31 | 1.65*** | 1.42-1.92 | 1.90 | 1.53-2.35 | | 0.69*** | | 0.59-0.80 | | 0.44*** | | 0.38-0.50 | | 0.70*** | | 0.63-0.77 | | 1.16*** | | 0.93-1.44 | |  |
| 0-5 years | 0.73*** | 0.52-1.01 | 1.15*** | 0.76-1.73 | 1.04*** | 0.85-1.28 | 1.20** | 0.90-1.59 | | 0.70*** | | 0.60-0.81 | | 0.51*** | | 0.46-0.57 | | 0.68*** | | 0.61-0.74 | | 1.43*** | | 1.19-1.71 | |  |
| Total number of observations 8516603 (with 47678 events of divorce) | | | | | | | | |  | |  | |  | |  | |  | |  | |  | |  | |  | |

Notes: Model adjusted for year, age composition, marital duration, educational composition and working/retirement status. Statistical significance levels: ***< 0.001, **< 0.01.

Table 2. The risk of divorce at age 60 or older by family complexity in the 3^rd^ generation interacted with marital duration, complementary log-log adjusted model, hazard ratios (reference marital duration within each family complexity category: 31+ years).

|  | Family complexity 3^rd^ generation | | | | | | | | | | | | | | | | | |
| --- | --- | --- | --- | --- | --- | --- | --- | --- | --- | --- | --- | --- | --- | --- | --- | --- | --- | --- |
|  | Joint grandchildren | | Joint grandchildren and she has grandchildren from previous unions | | Joint grandchildren and he has grandchildren from previous unions | | Joint grandchildren and both have grandchildren from previous unions | | She has grandchildren from previous unions | | He has grandchildren from previous unions | | Both have grandchildren from previous unions | | No grandchildren but couples has joint children | | No grandchildren but couples has stepchildren | |
| Marital duration | **HR** | **99% C.I.** | **HR** | **99% C.I.** | **HR** | **99% C.I.** | **HR** | **99% C.I.** | **HR** | **99% C.I.** | **HR** | **99% C.I.** | **HR** | **99% C.I.** | **HR** | **99% C.I.** | **HR** | **99% C.I.** |
| 31+ years | 1 |  | 1 |  | 1 |  | 1 |  | 1 |  | 1 |  | 1 |  | 1 |  | 1 |  |
| 26-30 years | 0.95 | 0.89-1.02 | 1.08 | 0.89-1.32 | 1.02 | 0.91-1.14 | 1.09 | 0.83-1.44 | 0.61*** | 0.53-0.71 | 0.46*** | 0.41-0.51 | 1.08 | 0.89-1.31 | 0.80*** | 0.75-0.86 | 0.38*** | 0.32-0.44 |
| 21-25 years | 0.83*** | 0.77-0.91 | 0.89 | 0.70-1.15 | 1.03 | 0.91-1.18 | 1.39** | 1.03-1.86 | 0.79*** | 0.70-0.90 | 0.56*** | 0.51-0.62 | 1.23** | 1.04-1.47 | 1.02 | 0.94-1.09 | 0.38*** | 0.33-0.43 |
| 16-20 years | 1.30** | 1.00-1.69 | 1.17 | 0.70-1.97 | 2.55*** | 1.84-3.54 | 1.97*** | 1.20-3.25 | 0.94 | 0.82-1.08 | 0.72*** | 0.66-0.79 | 1.47*** | 1.24-1.74 | 1.41*** | 1.29-1.54 | 0.45*** | 0.40-0.50 |
| 11-15 years | 0.95 | 0.59-1.52 | 1.04 | 0.49-2.21 | 1.74** | 0.98-3.11 | 1.54 | 0.74-3.20 | 1.09 | 0.96-1.24 | 0.78*** | 0.72-0.85 | 1.73*** | 1.47-2.04 | 1.51*** | 1.36-1.68 | 0.54*** | 0.49-0.60 |
| 6-10 years | 0.75 | 0.42-1.36 | 1.27 | 0.56-2.90 | 1.35 | 0.64-2.84 | 2.27** | 1.09-4.72 | 1.33*** | 1.17-1.51 | 0.87*** | 0.80-0.95 | 2.12*** | 1.80-2.50 | 1.54*** | 1.37-1.72 | 0.71*** | 0.64-0.78 |
| 0-5 years | 0.55* | 0.29-1.05 | 1.16 | 0.46-2.90 | 0.92 | 0.37-2.30 | 0.86 | 0.23-3.14 | 1.27*** | 1.11-1.45 | 0.85*** | 0.78-0.92 | 2.10*** | 1.78-2.48 | 1.44*** | 1.28-1.60 | 0.69*** | 0.63-0.76 |
| Total number of observations 7992904 (with 451888 events of divorce) | | | | | |  |  |  |  |  |  |  |  |  |  |  |  |  |

Notes: Model adjusted for year, age composition, marital duration, educational composition and working/retirement status. Statistical significance levels: ***< 0.001, **< 0.01.
